# Discovery of a New Acid

**Published:** 1800-06

**Authors:** 


### *Discovery of a new Acid.*

The celebrated chemiſt, Mr. Klaproth, of Berlin, has enriched chemiſtry with the diſcovery of a new acid, which he obtained by the analyſis of the honey ſtone, or melilithus. It is not a ſimple mineral acid, but rather of the nature of a vegetable acid; and according to the properties it ſhews, it muſt be conſidered as a peculiar modification of thoſe elements which conſtitute vegetable acids, and conſequently as a ſpecific vegetable acid, and an acid of its own. The properties it poſſeſſes are the following:

1. It cryſtallizes in ſmall fibrous and glomerated maſſes, and ſometimes in ſmall and ſhort columns. It does not appear to be able to cryſtallize at firſt, but gets the power of cryſtallizing by degrees, by imbibing more oxygen from the air.

2. Its taſte is ſweetiſh-ſour at firſt, but becomes afterwards a little bitter.

3. Poured upon a heated potſherd it quickly evaporates, ſpreading a dark grey ſmoke, which, however, does but little affect the ſmell; a ſmall quantity of yellowiſh light aſhes is left, which, moiſtened with water, are quite taſteleſs, and cauſe no change at all in blue colours.

4. Neutralized with kali, it forms a cryſtalline maſs of a fibrous, radiant texture.

5. Saturated with natron, or ſoda, it produces cubic cryſtallizations, partly diagonal ſingle priſms, partly concrete in a ſtellated manner.

6. The neutral ſalt which it produces with ammonia, exhibits ſmall hexagonal priſms, that loſe their tranſparency when expoſed to the air, and get a ſilver-white appearance.

7. Diſſolved in water, and dropped into lime-water, into a ſolution of burned barytes in water, it cauſes a white precipitation, which diſappears again by dropping to it ſome nitric acid.

8. A ſimilar precipitation takes place when it is dropped into a ſolution of acetated barytes (acetate de baryte), that alſo diſſolves again in nitric acid.

9. No precipitation followed with muriated barytes; but, ſoon after, ſome ſmall needle-line cryſtals became viſible in the mixture.

10. A ſolution of ſilver in nitric acid, remained clear in mixing it with the acid of the honey-ſtone.

11. A ſolution of nitrate of mercury mixed with it, lets fall a frequent precipitation of a white colour, which was diſſolved again by putting more nitric acid to the ſolution.

12. Iron diſſolved in nitric acid, formed a yellow precipitation with it, and was made ſoluble by the addition of muriatic acid.

13. A ſolution of the acetate of lead is much precipitated by it, but clearly diſſolved again by nitric acid.

14. With a ſolution of the acetate of copper, a verdigreaſe precipitation followed.

15. A ſolution of nitrate of copper ſuffered no change by it.

Theſe experiments ſhow, that this acid enters into a combination with ſeveral metallic calces, and that its affinity to them is greater than that of the acetous acid, though inferior to mineral acids. Similar effects are produced by its neutral ſalts, and the precipitation cauſed by them are in a great degree ſoluble.

As this acid is conſtituted by the carbon, oxygen, and hydrogen, and eaſily deſtroyed by fire; and as, at the ſame time, it agrees with none of the known acids in its properties and relation, it deſerves to be placed among the vegetable acids, as an acid of its own, to which the name of *Melilithick Acid* may be given.

The honey-ſtone, or Melilithus, from which it is obtained, is a foſſil found in Saxony, amongſt the ſtrata of bovey-coal (braunkohle) and was firſt deſcribed by Mr. Werner. It is of a honey-yellow colour, ſometimes darker, ſometimes lighter, and is always found in a cryſtallized octaedrous form, but ſeldom in a perfect ſtate. Its ſurface is generally ſmooth and ſhining, but ſometimes rough and eaten. It rarely is quite clear, but generally half tranſparent only, and it is ſoft, brittle, and eaſily reduced to a yellow grey powder. Its ſpecific weight is, according to Klaproth, 1,550. Mr. Klaproth was induced to ſubject this mineral to a new analyſis, in conſequence of the different opinions of chymiſts and mineralogiſts about its nature and conſtituent particles.* Some of them took it for a kind of amber, as Mr. Born; others, for a ſort of gypſum impregnated with petroleum. The chief particles are now, according

* Prof. Lampidius, of Freyberg, in Saxony, found in 100 parts:

| | |
|---|---|
| 85½ | carbon |
| 3½ | petroleum |
| 2 | ſilaceous earth |
| 5 | water of cryſtallization |
| 96 | |

Mr. Abick relates its conſtituent particles as follow:

| | |
|---|---|
| 40 | carbonic acid |
| 28 | water of cryſtallization |
| 16 | carbonic aluminous earth |
| 5 | benzoic aluminous earth |
| 5½ | benzoic acid |
| 5 | calx of iron |
| 2½ | reſin |
| 100 | |

cording to Mr. Klaproth, a peculiar acid combined with aluminous earth; and the proportion in 100 parts of it is, 16 parts of aluminous earth and 46 of acid, befides the water of cryftallization. *Crell's Annals, Vol. I.* 1800.

